# The Burden of Invasive Fungal Disease Following Chimeric Antigen Receptor T-Cell Therapy and Strategies for Prevention

**DOI:** 10.1093/ofid/ofae133

**Published:** 2024-03-13

**Authors:** Jessica S Little, Eleftheria Kampouri, Daniel Z Friedman, Todd McCarty, George R Thompson, Dimitrios P Kontoyiannis, Jose Vazquez, John W Baddley, Sarah P Hammond

**Affiliations:** Dana-Farber Cancer Institute, Harvard Medical School, Boston, Massachusetts, USA; Division of Infectious Diseases, Brigham and Women’s Hospital, Harvard Medical School, Boston, Massachusetts, USA; Infectious Diseases Service, Lausanne University Hospital and University of Lausanne, Lausanne, Switzerland; Section of Infectious Diseases and Global Health, The University of Chicago, Chicago, Illinois, USA; Division of Infectious Diseases, University of Alabama at Birmingham, Birmingham, Alabama, USA; Division of Infectious Diseases, University of California-Davis, Sacramento, California, USA; Department of Infectious Diseases, Infection Control and Employee Health, The University of Texas, M.D. Anderson Cancer Center, Houston, Texas, USA; Division of Infectious Diseases, Medical College of Georgia/Augusta University, Augusta, Georgia, USA; Division of Infectious Diseases, University of Maryland School of Medicine, Baltimore, Maryland, USA; Dana-Farber Cancer Institute, Harvard Medical School, Boston, Massachusetts, USA; Division of Infectious Diseases, Massachusetts General Hospital, Boston, Massachusetts, USA; Department of Medical Oncology, Massachusetts General Hospital Cancer Center, Boston, Massachusetts, USA

**Keywords:** antifungal prophylaxis, antifungal stewardship, CAR T-cell therapy, immunotherapy, invasive fungal disease

## Abstract

Chimeric antigen receptor (CAR) T-cell therapy is a novel immunotherapy approved for the treatment of hematologic malignancies. This therapy leads to a variety of immunologic deficits that could place patients at risk for invasive fungal disease (IFD). Studies assessing IFD in this setting are limited by inconsistent definitions and heterogeneity in prophylaxis use, although the incidence of IFD after CAR T-cell therapy, particularly for lymphoma and myeloma, appears to be low. This review evaluates the incidence of IFD after CAR T-cell therapy, and discusses optimal approaches to prevention, highlighting areas that require further study as well as future applications of cellular therapy that may impact IFD risk. As the use of CAR T-cell therapy continues to expand for hematologic malignancies, solid tumors, and most recently to include non-oncologic diseases, understanding the risk for IFD in this uniquely immunosuppressed population is imperative to prevent morbidity and mortality.

## Teaser

Chimeric antigen receptor (CAR) T-cell therapy is a rapidly expanding novel immunotherapy for treatment of hematologic malignancies. The characteristics of invasive fungal disease after CAR T-cell therapy are not well described, but the burden in published studies has been low.

## Graphical Abstract

**Graphical abstract:**
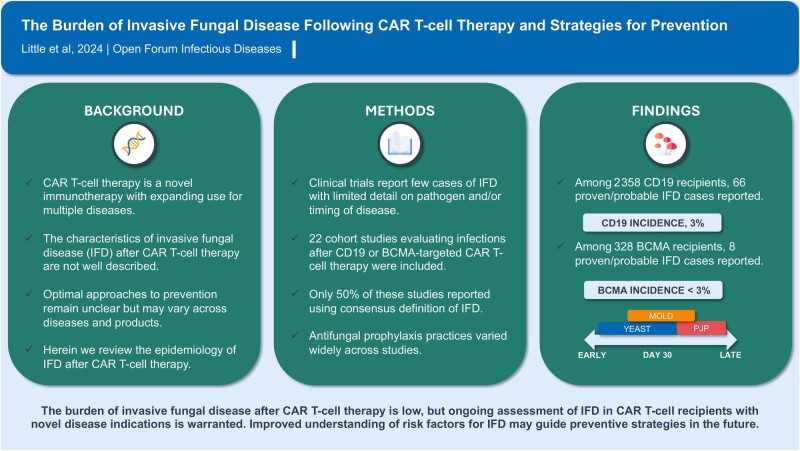

Chimeric antigen receptor (CAR) T-cell therapies are a novel class of immunotherapy that genetically engineers patients’ T cells to target specific disease-related antigens enabling rapid killing of dysregulated cells. This immunotherapy has revolutionized the management of relapsed/refractory (R/R) B-cell and plasma cell hematologic malignancies inducing durable responses in patients facing dire prognoses. Several products targeting the CD19 tumor antigen are currently approved for R/R B-cell malignancies (Table 1) [1–9]. More recently, 2 B-cell maturation antigen (BCMA)–targeted products were approved for R/R multiple myeloma (MM) (Table 1) [10–13]. Beyond the approved products, a multitude of trials are ongoing [14], and CAR T-cell use is rapidly expanding for hematologic malignancies, solid tumors, and non-oncological indications such as autoimmune diseases and infections [15–20]. Importantly, the place of these therapies is evolving, shifting to an earlier line of treatment in populations of patients with less refractory disease [6, 21–23]. As a result, the pool of CAR T-cell recipients continues to grow.

**Table 1.** Commercially Available Chimeric Antigen Receptor T-Cell Products and Indications

CAR T-Cell Product	Indication
CD19-targeted CAR T-cell products	
Tisagenlecleucel (Kymriah; Novartis)	B-ALL, large B-cell lymphoma, and follicular lymphoma
Axicabtagene ciloleucel (Yescarta; Kite/Gilead)	Large B-cell lymphoma and follicular lymphoma
Brexucabtagene autoleucel (Tecartus; Kite/Gilead)	B-ALL and mantle cell lymphoma
Lisocabtagene maraleucel (Breyanzi; Juno/BMS)	Large B-cell lymphoma
BCMA-targeted CAR T-cell products	
Idecabtagene vicleucel (Abecma; Celgene/BMS)	Relapsed/refractory multiple myeloma
Ciltacabtegene autoleucel (Carvykti; Janssen/Legend)	Relapsed/refractory multiple myeloma

Abbreviations: B-ALL, B-cell acute lymphoblastic leukemia; BCMA, B-cell maturation antigen; CAR, chimeric antigen receptor.

CAR T-cell therapies are effective, but these potent “living drugs” come at the price of unique toxicities and a high net burden of immunosuppression [24–29]. Accordingly, infections are common and the key determinant of nonrelapse mortality [26, 30]. Invasive fungal disease (IFD) is a morbid complication of immunosuppressive therapy. It is well described following hematopoietic cell transplantation (HCT) [31–33], yet an understanding of the incidence and risk factors for IFD following CAR T-cell therapy remains limited [34]. This is in part due to a lack of standardized reporting of opportunistic infections in large-scale clinical trials [35–37]. Furthermore, real-world studies are limited by small numbers, inconsistent definitions of IFD, and varying approaches to prophylaxis. Few studies have described IFD after CAR T-cell therapy with attention to pathogen type, timing, management, and outcomes, and no individual risk factors for IFD in this setting have been presented [38].

Accurately assessing IFD epidemiology is a prerequisite to evidence-based strategies to reduce associated morbidity and mortality in an expanding and uniquely immunocompromised population of CAR T-cell therapy recipients. Importantly, a one-size-fits-all approach may not be suitable as different CAR T-cell targets and patient populations can have distinct risks. Infectious diseases teams should play a key role in answering these questions and optimizing prevention and management of IFD after CAR T-cell therapy while ensuring the promotion of diagnostic and antifungal stewardship. Herein we review the epidemiology of fungal infections after CAR T-cell therapy, current preventive strategies, and unmet needs in the field.

## NET STATE OF IMMUNOSUPPRESSION: TREATMENT AND HOST-RELATED RISK FACTORS FOR INVASIVE FUNGAL DISEASE

**Figure 1.**
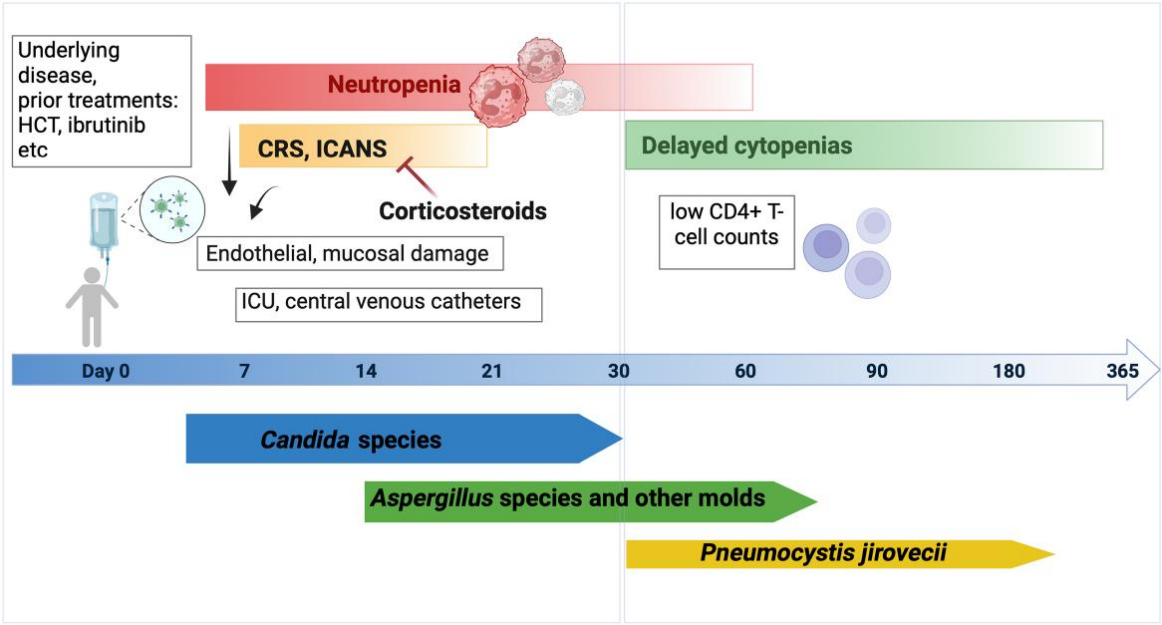
Treatment and host-related risk factors and timing of invasive fungal disease (IFD) after chimeric antigen receptor (CAR) T-cell therapy. A variety of host factors may impact the risk for IFD after CAR T-cell therapy and play a role in the timing of various fungal diseases. Invasive yeast infections tend to occur early, in the first 30 d after CAR T-cell therapy, invasive mold infections occur both early and late (after day 30), and cases of *Pneumocystis* pneumonia primarily occur after day 30, with some cases reported even beyond 1 y after CAR T-cell therapy. This conceptual model for this figure was adapted from Tomblyn et al [40]. Created with Biorender.com. Abbreviations: CRS, cytokine release syndrome; HCT, hematopoietic cell transplantation; ICANS, immune effector cell–associated neurotoxicity; ICU, intensive care unit.

Autologous CAR T cells are produced after patients undergo apheresis of T cells. Cells then undergo laboratory-based genetic engineering to express chimeric antigen receptors targeting specific disease/tumor antigens. Patients are treated with lymphodepleting chemotherapy to create a favorable environment for the immune cells, prior to reinfusion. CAR T-cell therapy recipients are at increased risk for infection due to a plethora of factors including the underlying malignancy and prior treatments such as HCT; lymphodepleting chemotherapy and other bridging chemo-immunotherapies; post–CAR T-cell acute toxicities including cytokine release syndrome (CRS) and immune effector cell (IEC)–associated neurotoxicity syndrome (ICANS) and their management with immunomodulatory treatments; neutropenia, which can be prolonged in nature; and “on target, off tumor” effects leading to long-lasting B-cell aplasia and antibody deficiencies (Figure 1) [27, 34, 39]. Other infections, such as those due to bacteria and viruses, that develop after CAR T-cell therapy may also modify the risk for IFD, but this association has not yet been studied. Although the specific role of these risk factors in the occurrence of IFD has not been systematically assessed in the setting of CAR T-cell therapy, these factors can increase risk of IFD, directly or indirectly, and should be carefully considered when planning preventive strategies.

### Host Factors: Underlying Malignancy and Prior Treatments

Several oncologic factors have been associated with increased overall risk for infection after CAR T-cell therapy: diagnosis of B-cell acute lymphoblastic leukemia (B-ALL) (compared to lymphoma) [41], increasing lines of prior antitumor therapy [41–43], and previous allogeneic HCT [44]. The underlying disease and prior treatments are important determinants of IFD risk in patients with hematological malignancies in general, and likely play a role in IFD risk for CAR T-cell recipients. Determining differential risk of IFD between populations is limited by heterogeneity of patients and treatment regimens. Notably, patients with acute myeloid leukemia (AML) and undergoing allogeneic HCT are traditionally considered at higher risk for IFD compared to patients with B-ALL, B-cell lymphoma, and MM [45]. R/R B-ALL, lymphoma, and MM patients receiving CAR T-cell therapy are likely at a higher risk for IFD compared to the overall disease groups, though comparative data are scarce [45]. Allogeneic HCT, which is more frequently utilized for R/R B-ALL patients than B-cell lymphoma or myeloma patients, has long been associated with increased IFD risk, and may independently impact post–CAR T-cell therapy risk for IFD [45, 46]. Importantly, the impact of HCT on IFD risk in CAR T-cell therapy recipients is likely influenced by the time from HCT, the status of disease post-HCT, complications, and treatments (eg, graft-versus-host disease). Finally, specific targeted antineoplastic therapies such as Bruton tyrosine kinase inhibitors (eg, ibrutinib), administered prior to CAR T-cell therapy, are associated with invasive mold infections and may contribute to post–CAR T-cell therapy IFD risk [37, 47, 48].

### Treatment Factors: Neutropenia

The impact of severe, prolonged neutropenia on risk of IFD is well established [49–51]. Severe neutropenia (<500 cells/μL) develops in >90% of CAR T-cell therapy recipients after lymphodepleting chemotherapy but is typically less prolonged than after HCT, with a median duration of 9 days [25, 28, 52, 53]. A biphasic temporal course of neutropenia is frequently observed (50% of patients) with intermittent recovery of neutrophils around week 3 and a second trough (<1000 cells/μL) 2 months after infusion, while an aplastic phenotype with continuous severe neutropenia (<500 cells/μL) for at least 14 days is observed in one-quarter of patients [25, 26]. While the first neutropenic phase is strongly linked with lymphodepleting chemotherapy and compounded by immune dysregulation and impaired hematopoietic function, the second phase is independent of any systemic myelotoxic therapy and likely immune-mediated though the exact mechanism is unknown [53, 54]. Improved understanding of the impacts of prolonged or late neutropenia on infection and in particular IFD risk is needed.

### Treatment Factors: CRS, ICANS, and IEC-Associated Hemophagocytic Lymphohistiocytosis–Like Syndromes

Cytokine release syndrome [41, 55] and ICANS [41, 56–58], and the corticosteroids utilized for their management [26, 42, 57, 59, 60], are important independent risk factors for post–CAR T-cell therapy infections. CRS, which occurs in 57%–93% of CAR T-cell recipients, and ICANS, which occurs in 20%–70% of CAR T-cell recipients, are associated with profound immune dysregulation leading to endothelial damage and loss of mucosal integrity. These entities are both treated with immunosuppressive treatments including corticosteroids and tocilizumab, and may require invasive measures for the management of critically ill patients (central venous catheters, mechanical ventilation) [61]. IEC-associated hemophagocytic lymphohistiocytosis is rare (<5%) and is characterized by a more severe hyperinflammatory syndrome often requiring very prolonged immunosuppressive therapy [61–63]. These toxicities may increase the risk of IFD after CAR T-cell therapy, particularly in their most severe forms with associated prolonged or high-dose corticosteroids—a major driver of IFD risk in hematologic malignancies [64]. The cumulative dose of corticosteroids and its impact on IFD after CAR T-cell therapy represents an important area for future investigation. The effect of tocilizumab on IFD risk is less clear, with some reports describing a higher incidence of IFD in patients with severe coronavirus disease 2019 receiving tocilizumab, while several large studies show no association in the setting of autoimmune diseases [65–68].

### Treatment Factors: Late Hematologic Toxicities

Delayed cytopenias remain a major complication beyond the first month [24, 53]. Cellular immunity is also durably impaired in CD19 CAR T-cell recipients; CD4^+^ T-cell counts decrease after infusion and may remain low, with a median of 155 cells/μL at 1 year [57] and <200 cells/μL in half of patients at 18 months post-infusion [59]. The durable impairment of cellular immunity with slow recovery of CD4^+^ T-cell counts may be associated with increased IFD risk, as manifested by the cases of late *Pneumocystis jirovecii* occurring >3 months post–CAR T-cell infusion [38, 57, 59]. The long-lasting B-cell aplasia, hypogammaglobulinemia, and specific antibody deficiencies due to “on target, off tumor” effects further increase overall infection risk, and while the link with IFD is more insidious, they could further indirectly impact cellular immunity through the complex interplay with T cells [47]. Finally, while CAR T-cell therapies induce durable remissions in a high proportion of patients, relapse also can occur in >50% of patients, though response rates may vary by product and disease [69]. Response to treatment and the need for additional antitumor therapies impact IFD risk [70].

## EPIDEMIOLOGY OF INVASIVE FUNGAL DISEASE AFTER CAR T-CELL THERAPY

Phase 1/2 trials for R/R disease across multiple CD19-targeted products have reported zero invasive fungal infections [2, 3, 5, 9, 71]. Long-term follow-up studies (>2 years) have since provided updates with an incidence of 3% (2 *Candida* spp infections and 1 invasive pulmonary aspergillosis [IPA]) in the axicabtagene ciloleucel cohort, and 4% in the tisagenlecleucel cohort (2 *Candida* spp infections; 1 IPA; and 2 *Pneumocystis jirovecii* pneumonia [PJP]) without detail on timing or clinical outcomes [71, 72]. One major study of lisocabtagene maraleucel with a median of 19 months of follow-up reported only 2 fungal infections (1%; 1 candiduria; 1 invasive candidiasis) [9]. More recent phase 3 trials evaluating CD19 products as second-line therapy versus standard of care have also reported no fungal infections. Among BCMA products, phase 1 studies of idecabtagene vicleucel and ciltacabtagene autoleucel reported no fungal infections [11, 13]. In phase 2 studies, 11 fungal infections among 128 patients within 24 months were reported in 1 study. However, it was not specified whether these infections were invasive or what pathogens were involved [12]. In larger phase 3 studies of BCMA-targeted products, only a few cases of IFD were reported with an incidence in both studies <1% (1 bronchopulmonary aspergillosis and 1 *Candida* sepsis in the idecabtagene vicleucel study; 1 case of PJP in the ciltacabtagene autoleucel study). Given the low number of cases of IFD with limited clinical description reported in clinical trials thus far, we focus here on IFD reported in 22 cohort studies evaluating infections after CAR T-cell therapy. Case reports, while useful for understanding the clinical course of IFD in this population, were not included in this analysis, given the challenges in evaluating the disease incidence without an understanding of the overall denominator of patients treated [64].

Risk of IFD ranged from 0 to 15% in CD19 CAR T-cell recipients and from 0 to 8% in BCMA CAR T-cell recipients in individual studies, although standard definitions were not always used, and noninvasive cases were at times included, potentially leading to elevated estimates of IFD. Among 22 studies evaluating infections after CD19/BCMA CAR T-cell therapy, only 11 (50%) studies reported using the European Organisation for Research and Treatment of Cancer/Mycoses Study Group Education and Research Consortium (EORTC/MSGERC) Consensus Definitions for IFD. Of these, 2 included noninvasive cases, and 1 included 3 cases of possible IFD (Table 2) [26, 59, 73]. Antifungal prophylaxis practices varied widely (Table 3). Most centers used fluconazole, others used none, and some used mold-active prophylaxis for specific risk populations. Follow-up was heterogenous, ranging from 30 days to >2 years. In this review, we focus on proven/probable cases of IFD and exclude cases of mucosal candidiasis and pulmonary nodules/consolidation without supporting mycological evidence (EORTC “possible” IFD) to accurately assess the true incidence of IFD and characterize the timing and clinical presentation [74, 75].

**Table 2.** Definitions of Invasive Fungal Disease in Published Studies Evaluating Infections after Chimeric Antigen Receptor T-Cell Therapy

Definitions of Invasive Fungal Disease	No.
Reported using EORTC/MSGERC Consensus Definitions of Invasive Fungal Disease and included only invasive cases	8
Reported using EORTC/MSGERC Consensus Definitions of Invasive Fungal Disease and included noninvasive cases	2
Reported using EORTC/MSGERC Consensus Definitions of Invasive Fungal Disease and included possible cases of IFD	1
Did not report using EORTC/MSGERC Consensus Definitions of Invasive Fungal Disease	10

Abbreviations: EORTC/MSGERC, European Organisation for Research and Treatment of Cancer/Mycoses Study Group Education and Research Consortium; IFD, invasive fungal disease.

**Table 3.** Incidence of Invasive Fungal Disease in CD19 and B-Cell Maturation Antigen Chimeric Antigen Receptor T-Cell Recipients

Study, First Author	No. of Patients	Disease	Follow-up	Anti-yeast Prophylaxis	Mold-active Prophylaxis	Invasive Fungal Disease	Invasive Yeast Infection	Invasive Mold Infection	Other Fungal Disease
CD19 CAR T-cell therapy									
Hill 2018 [41]	133	NHL 47% ALL 35% CLL 18%	100 d	Fluconazole	None	8 (6)	4 (3)	3 (2)	1 (1)
Park 2018 [55]	53	ALL	180 d	Micafungin	Variable^a^	5 (9)	1 (2)	4 (8)	0 (0)
Cordeiro 2020 [76]	86^b^	ALL 50% ALL 30% CLL 20%	90 d to 28 mo (median)	NR	NR	3 (6)	0 (0)	2 (4)	1 (2)
Haidar 2020 [77]	59	Pt 1: ALL Pt 2: Hairy cell leukemia	5 mo	Fluconazole	Mold-active azole with prolonged neutropenia	NR	NR	2 (3)	NR
Vora 2020 [44]	83	ALL 98%	100 d	Fluconazole	Mold-active azole with prior IFI	1 (1)	0 (0)	1 (1)	0 (0)
Logue 2021 [57]	85	NHL	1 y	Fluconazole	None	2 (2)	1 (1)	1 (1)	0 (0)
Wudhikarn 2020 [60]	60	DLBCL	1 y	Fluconazole	Mold-active azole with prolonged steroids	2 (3)	0 (0)	1 (2)	1 (2)
Zhu 2021 [58]	113	ALL 66% NHL 34%	180 d	NR	NR	4 (4)	2^c^ (2)	2 (2)	0 (0)
Baird 2021 [59]	41	NHL	1 y	Fluconazole	None	6^d^ (15)	2 (5)	1 (2)	3 (7)
Beyar-Katz 2022 [56]	60	DLBCL	1 mo	Fluconazole	None	0 (0)	0 (0)	0 (0)	0 (0)
Wittman Dayagi 2021 [78]	88	ALL 43% NHL 57%	60 d	None	None	1 (1)	0 (0)	1 (1)	0 (0)
Mikkilineni 2021 [42]^e^	72	ALL 69% NHL 31%	30 d	Micafungin	Mold-active azole^f^	0 (0)	0 (0)	0 (0)	0 (0)
Little 2022 [38]	280	NHL	1 y	None	None	8 (3)	2 (0.7)	3 (1)	3 (1)
Rejeski 2022 [26]	248	NHL	90 d	Fluconazole	Variable^g^	24^h^ (10)	19^f^ (8)	4 (2)	1 (0.4)
Czapka 2023 [79]	73	NHL 97%	2 y	Fluconazole or micafungin	Mold-active azole with prolonged neutropenia or high-dose steroids	5 (7)	4 (5)	1 (1)	0 (0)
Mercadal 2023 [80]	48	NHL	180 d	Fluconazole	None	2 (4)	1 (2)	1 (2)	0 (0)
BCMA CAR T-cell therapy									
Kambhampati 2022 [81]	55	MM	1 y	Fluconazole	None	3 (5)	NR^i^	2 (4)	NR
Logue 2022 [73]	52	MM	100 d	Fluconazole	Mold-active azole with prolonged steroids or prior IFI	3 (6)	NR^j^	NR	NR
Wang 2021 [52]	40	MM	16 mo (median)	NR	NR	3 (8)	NR^k^	NR	NR
Josyula 2022 [82]	32	MM	180 d	Fluconazole	None	2 (6)	0 (0)	2 (6)	0 (0)
Mohan 2022 [83]	26	MM	9 mo (median)	Fluconazole	Mold-active azole with high-dose steroids	0 (0)	0 (0)	0 (0)	0 (0)
Mikkilineni 2021 [42]	24^e^	MM	30 d	Micafungin	Mold-active azole^f^	0 (0)	0 (0)	0 (0)	0 (0)
Little 2023 [84]	99	MM	1 y	Fluconazole with high-dose steroids or prolonged neutropenia	None	3 (3)	1 (1)	2 (2)	0 (0)

Abbreviations: ALL, acute lymphoblastic leukemia; BCMA, B-cell maturation antigen; CAR, chimeric antigen receptor; CLL, chronic lymphocytic leukemia; DLBCL, diffuse large B-cell lymphomaI; IFD, invasive fungal disease; MM, multiple myeloma; NHL, non-Hodgkin lymphoma; NR, not reported.

^a^Nine patients received voriconazole or posaconazole; reasons not reported.

^b^Only 54 patients were assessed for infection.

^c^Not specified if invasive candidiasis; 1 case included pulmonary site.

^d^Five reported cases of oropharyngeal candidiasis and 1 cutaneous candidiasis were excluded from this analysis as well as 3 cases of “pulmonary infection” characterized by pulmonary consolidation or nodule on imaging not attributed to bacterial or viral causes as these do not meet European Organisation for Research and Treatment of Cancer/Mycoses Study Group Education and Research Consortium (EORTC/MSGERC) criteria for invasive fungal infection.

^e^Combined study of multiple products: 20 adult patients and 52 pediatric patients received CD19-directed products; 24 patients received BCMA-directed products.

^f^Reasons for prophylaxis not specified.

^g^Antifungal prophylaxis varied across centers including mold-active azoles in multiple centers.

^h^Includes multiple pulmonary isolates of *Candida* (a nonsterile space) and only used positive microbiologic specimens for diagnosis rather than EORTC definitions for invasive disease.

^i^One non-mold infection reported; not specified.

^j^Reported 1 possible fungal pneumonia and 2 possible fungal skin/soft tissue infections.

^k^Fungal infection type not specified.

**Figure 2.**
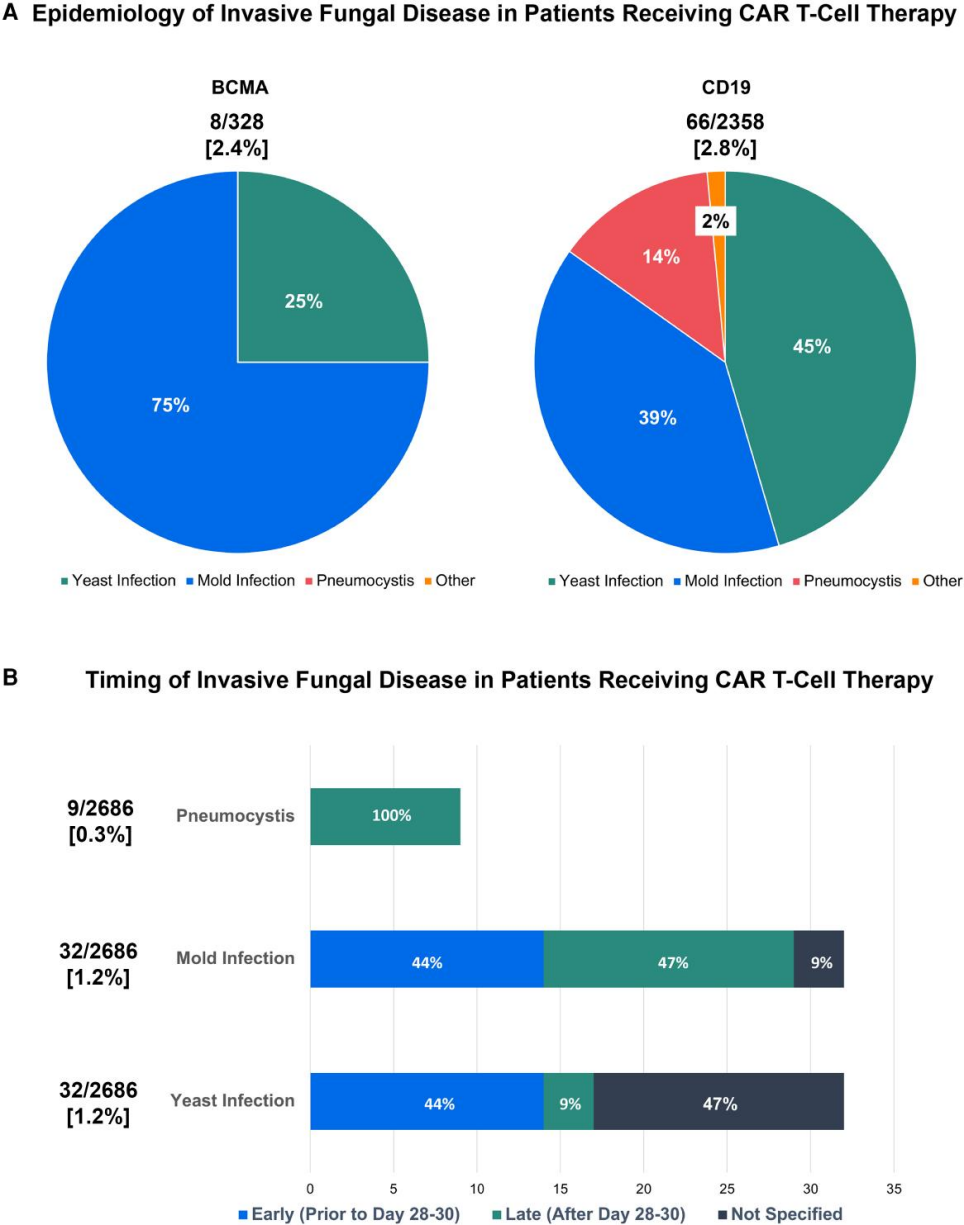
Characteristics of invasive fungal disease (IFD) after chimeric antigen receptor (CAR) T-cell therapy. *A*, Overall breakdown of invasive yeast infections, invasive mold infections, *Pneumocystis jirovecii* pneumonia (PJP), and other IFDs among patients receiving CD19-directed and B-cell maturation antigen (BCMA)–directed CAR T-cell therapy. Among CD19 CAR T-cell recipients, invasive yeast infections were most common (45%), followed by invasive mold infections (39%), then PJP (14%), and 1 case of coccidioidomycosis (2%). Among BCMA CAR T-cell recipients, invasive mold infections were the most frequent IFD (75%) with invasive yeast infections comprising only 25% of cases. There were no cases of PJP or other IFDs. *B*, Timing of IFDs after CAR T-cell therapy. Yeast infections primarily occurred early (prior to day 30) in 44% of cases, although timing was not reported in 47% of cases. Mold infections occurred both early (44%) and late (47% after day 30). PJP occurred only after day 30 in all cases.

Across 16 studies evaluating 2358 CD19 CAR T-cell recipients, there were 71 cases of IFD reported, of which 66 were identified as proven/probable (overall incidence, 2.8%). Of the 66 proven/probable cases, 45% were invasive yeast infections, 39% invasive mold infections (IMIs), 14% PJP, and 1 coccidioidomycosis. Seven studies evaluating BCMA CAR T-cell recipients (n = 328) described 14 IFD cases, of which 8 were proven/probable (overall incidence, 2.4%). The 8 proven/probable IFD cases in BCMA recipients consisted primarily of IMIs (75%) and 25% due to yeast infections, and no PJP cases were documented (Figure 2*A*). Among BCMA and CD19 CAR T-cell recipients, yeast infections tended to occur early (day 0–30) while mold infections were evenly split between early and late presentations (after day 30; Figure 2*B*). PJP only occurred after day 30, which is likely a reflection of universal prophylaxis use for at least 6 months, but speaks to the immunologic deficits that can persist even beyond 1 year [38, 57, 59]. Endemic mycoses and cryptococcosis have been rarely reported, with only 1 case of coccidioidomycosis identified [76, 85].

### Invasive Yeast Infections After CAR T-Cell Therapy

Assessment of invasive yeast infections after CAR T-cell therapy has been limited by the inclusion of mucosal candidiasis and nonsterile *Candida* cultures (respiratory or skin) without correlative evidence of invasive disease [26, 59]. Among CD19 CAR T-cell recipients, the incidence of invasive yeast infections ranged from 0 to 8%, though the study reporting 8% included *Candida* cultures of nonsterile respiratory samples. Importantly, the majority of CD19 studies (11/16) reported a low incidence of invasive yeast infections (0–2%). While most included centers did administer anti-yeast prophylaxis during the period of neutropenia, 2 centers without yeast prophylaxis also reported low incidence of 0–0.7%, suggesting that this is a reasonable approach in certain settings [38, 78]. For BCMA CAR T-cell recipients, the epidemiology of invasive yeast infections is less well described with 3 studies not specifically reporting on the type of IFD, 3 studies reporting no yeast infections, and 1 study reporting 1 yeast infection (1%) [73, 81–84, 86, 87].

**Figure 3.**
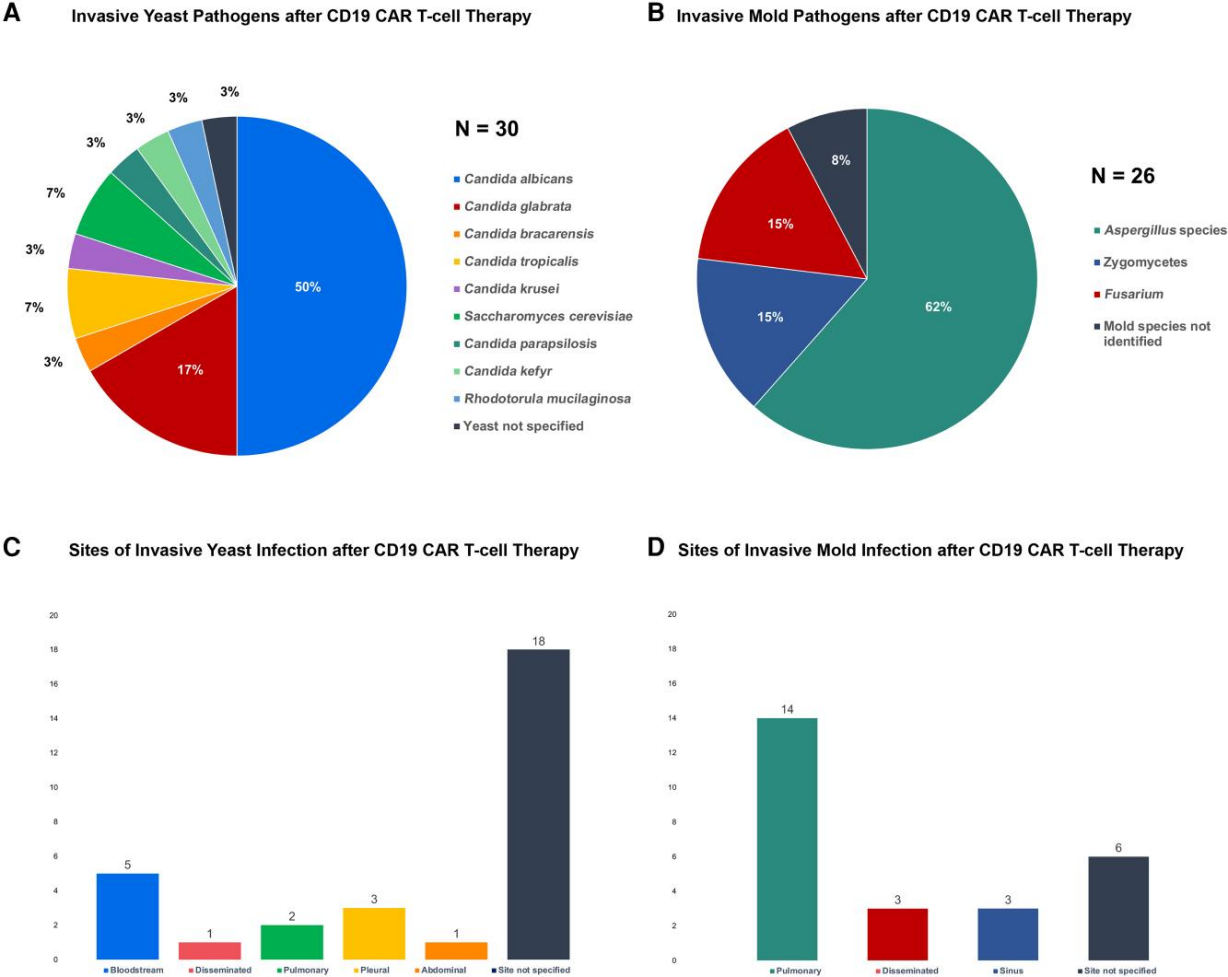
Invasive yeast and invasive mold infections after CD19 chimeric antigen receptor (CAR) T-cell therapy. The specific pathogens identified among invasive yeast and mold infections (*A* and *B*) in CD19 CAR T-cell recipients, as well as the reported sites of infection (*C* and *D*), are shown.

Across CD19/BCMA CAR T-cell studies, 32 of 2686 patients (1.2%) developed proven/probable invasive yeast infections with invasive candidiasis comprising the majority of the infections (89%). *Candida albicans* infections were most frequent (50% for CD19/BCMA), and *Nakaseomyces glabrata* (formerly *Candida glabrata*) was also commonly identified (Figure 3). Yeast infections occurred early (prior to day 30) in 82% of cases and often in the setting of CRS or ICANS (Figure 2*B*) [38, 41, 55]. Infection sites in CD19 CAR T-cell recipients included bloodstream (n = 5; Figure 3*C*), disseminated (n = 1), pulmonary (n = 2), pleural (n = 3), abdominal (n = 1), and 18 isolates from 1 study where a positive culture was reported without a description of the involved site [26]. The 2 BCMA yeast cases included a case of *Candida albicans* peritonitis and 1 yeast infection without pathogen/site identified.

Invasive yeast infections are infrequent after CAR T-cell therapy, which is not necessarily related to use of anti-yeast prophylaxis during neutropenia since several studies report low incidence without the use of prophylaxis [38, 57, 79]. Breakthrough cases have occasionally been reported with resistant organisms in studies where prophylaxis was utilized. Yeast infections typically occur within the first 30 days (spanning the period of neutropenia) and may be more frequent in those with CRS or ICANS. Additional studies are needed to better describe the epidemiology of yeast infections after BCMA CAR T-cell therapy as data remain limited.

### Invasive Mold Infections After CAR T-Cell Therapy

The incidence of IMI in CD19 CAR T-cell recipients ranges from 0 to 8% (Table 3). Earlier studies evaluating clinical trial populations and those including B-ALL patients appeared to have higher incidence of IMI [41, 55, 76, 77]. All studies published from 2020 onward that primarily include real-world commercial CAR T cells for B-cell lymphoma have demonstrated a low incidence of IMI of 0–2%. This shift may reflect advances in the management of acute toxicities including CRS/ICANS and increasing use of CAR T-cell therapy as an earlier line of therapy. However, it is possible that IFD cases may be missed with decreasing rates of autopsy and the known limitations of antifungal diagnostic testing [46, 88]. BCMA CAR T-cell studies evaluating infections are fewer in number and have predominantly short follow-up durations but demonstrated an incidence of IMI of 0–6% (Table 3). Overall, mold-active prophylaxis is typically reserved for high-risk patients. However, centers with no mold-active prophylaxis also report a low rate of IMI, raising the question of whether mold-active prophylaxis is actually indicated (Table 3) [38, 56, 57, 78, 80, 81, 84]. Thus far, some breakthrough infections have been reported with rare/resistant pathogens (*Fusarium* spp, n = 1; *Cunninghamella* spp, n = 1) in patients receiving mold-active azoles [44, 77].

**Figure 4.**
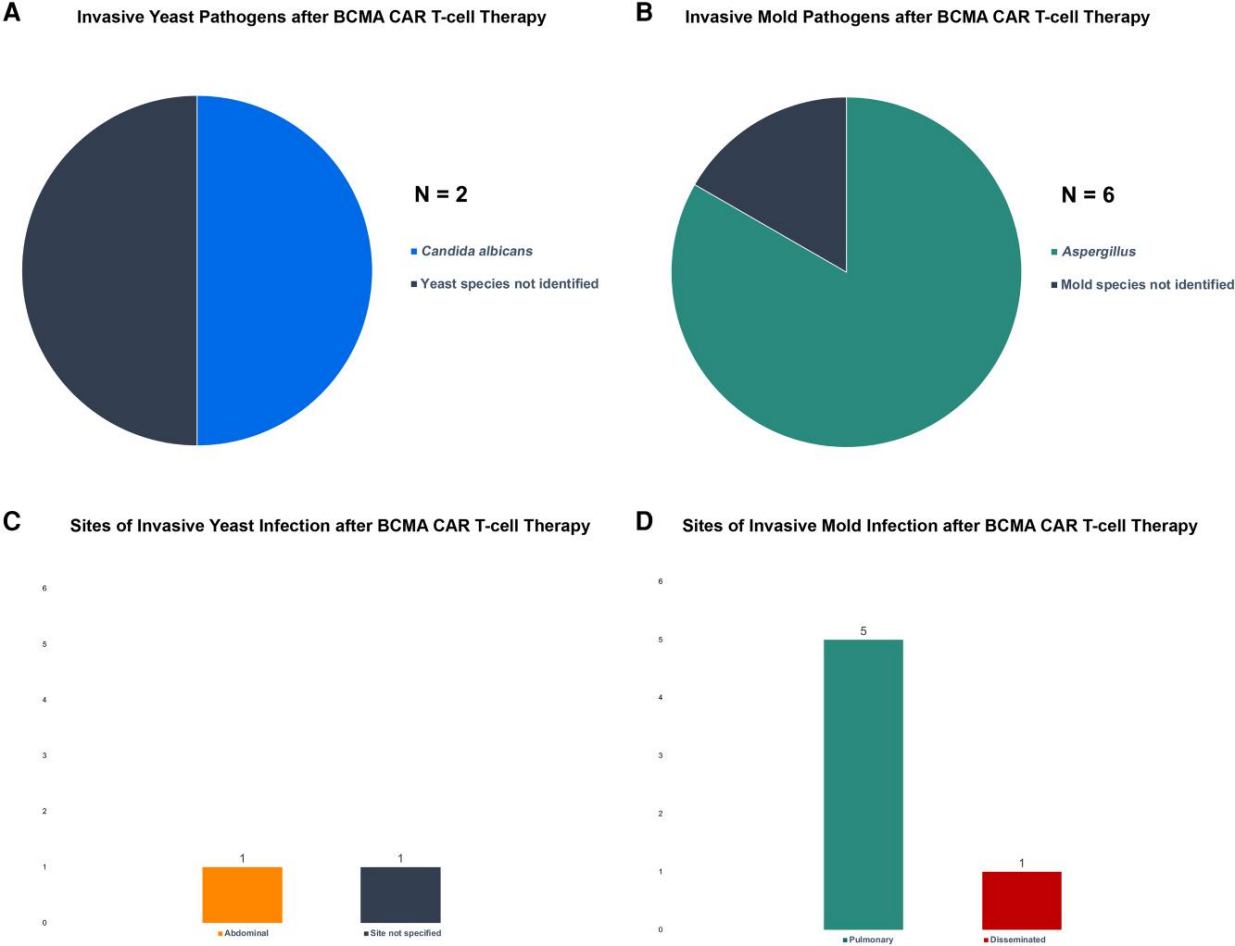
Invasive yeast and invasive mold infections after B-cell maturation antigen (BCMA) chimeric antigen receptor (CAR) T-cell therapy. The specific pathogens identified among invasive yeast and mold infections (*A* and *B*) in BCMA CAR T-cell recipients, as well as the reported sites of infection (*C* and *D*), are shown.

In the evaluated studies, 32 of 2686 patients (1.2%) had proven/probable IMI, with *Aspergillus* as the predominant pathogen in 64%. In CD19 CAR T-cell recipients, mucormycosis and fusariosis each comprised 15% of IMI cases (Figure 3*B*). In 8%, a genus was not identified. In BCMA CAR T-cell recipients, no rare molds have been reported, but in 2 of 6 cases (33%) the genus was not able to be identified. The most frequently involved site was pulmonary in both CD19 and BCMA CAR T-cell recipients (54% and 83% respectively; Figures 3*D* and 4*D*). Other infection sites in CD19 recipients included disseminated (n = 3) and sinus (n = 3) as well as 6 cases where sites were not reported (Figure 3*D*). One disseminated mold infection occurred in a BCMA CAR T-cell recipient (Figure 4*D*).

IMI is rare after CAR T-cell therapy despite a high net state of immunosuppression, early neutropenia related to lymphodepleting chemotherapy, CRS/ICANS with associated with additional immunosuppressive therapy, and delayed hematologic toxicity including late neutropenia and impaired cell-mediated immunity [28, 57, 59, 89–92]. Differences in risk between disease groups may also play a role in varying incidences of IMI across studies. In the future, comparative studies with larger populations are needed to clarify these nuances.

### *Pneumocystis* Pneumonia After CAR T-Cell Therapy

*Pneumocystis jirovecii* prophylaxis has been largely adopted following CAR T-cell therapy given similar immunologic deficits to HCT, with most centers administering prophylaxis for 6–12 months and typically utilizing CD4 T-cell counts <200 cells/μL to guide duration [34]. Few cases of PJP have been reported following CAR T-cell therapy, which is likely related to use of prophylaxis as well as variable follow-up in most published studies. We identified 9 cases of PJP in CD19 CAR T-cell recipients after cessation of prophylaxis, including several that occurred after 1 year [26, 38, 41, 59, 60]. No cases of PJP have been reported among BCMA CAR T-cell recipients, though it remains unclear if this is related to a distinct risk, differences in prophylaxis practices, or the small number of studies with limited follow-up. While persistent and profound B-cell aplasia is a recognized “on target, off tumor” effect of CAR T-cell therapy, T-cell depletion and long-term deficits in cell-mediated immunity are present but are poorly understood. The impact of these deficits on infection risk beyond 1 year requires further study and may aid in identifying patients at higher risk for late infections including PJP [57, 59].

## PREVENTION OF INVASIVE FUNGAL DISEASE: GUIDING PRINCIPLES

While IFD continues to be associated with excess mortality, the approach to management and prevention in the immunocompromised populations has evolved substantially. In the 1990s to early 2000s, several landmark trials established the benefit of fluconazole in reducing the incidence of invasive candidiasis and death after HCT [93–95]. Widespread use of fluconazole prophylaxis following HCT was adopted, although preemptive treatment remained an effective strategy at many centers [51]. However, shifting epidemiology with recent increases in the incidence of IMIs has driven further changes in prophylactic strategies [33, 96–98]. Two pivotal trials in 2007 demonstrated the benefit of posaconazole over fluconazole in preventing aspergillosis in patients undergoing HCT or receiving remission-induction chemotherapy for AML [50, 99]. Currently, mold-active azoles are utilized for prophylaxis in patients with AML and those undergoing HCT. While clinical trial data have not explored the utility of mold-active azoles in a broader immunosuppressed population, prophylaxis has been adopted at many centers for indications outside of HCT/AML based on rates of breakthrough fungal infection [100].

The potential disadvantages of antifungal prophylaxis must also be considered. Antifungal use may shift fungal epidemiology and impact the incidence of rare/resistant species, as demonstrated by increased rates of *Pichia kudriavzevii* (formerly *Candida krusei*) in the years following the institution of routine fluconazole prophylaxis in many centers [101–103]. Increased reports of invasive mucormycosis in patients receiving voriconazole prophylaxis, also suggest that antifungal exposure may impact the spectrum of fungal disease [104–108]. While posaconazole and isavuconazole do target the Mucorales, it remains to be seen whether rising rates of emerging, resistant fungal pathogens such as *Fusarium* and *Scedosoporium*/*Lomentospora* spp could be related to the application of broader-spectrum agents for routine prophylaxis [109, 110]. Beyond these rare mold species, increasing antifungal resistance globally including azole-resistant *Aspergillus* is in part driven by healthcare-associated and agricultural use of azoles and represents a serious public health risk [109, 111]. Subtherapeutic levels of azoles in vivo could exacerbate this problem in centers that do not utilize therapeutic drug monitoring [112, 113]. Even without documented azole resistance, breakthrough IFD (bIFD) is more challenging to treat, typically requiring a switch to liposomal amphotericin B with associated toxicities that can be difficult to tolerate in critically ill patients, and presents challenges to long-term and outpatient administration and monitoring [114–116]. Finally, while azoles have largely been demonstrated to be tolerable, adverse effects and serious drug–drug interactions can occur and should be balanced carefully against the potential benefits [117–119].

A balanced approach with an emphasis on antifungal stewardship should be employed when expanding use of antifungal prophylaxis in novel immunosuppressed populations. Published studies on bIFD suggest that prophylaxis strategies cannot prevent all fungal infections. In fact, a recent publication showed that bIFD was common and occurred in 7% of patients [100]. Thus, consideration of the baseline incidence of IFD in a certain population can inform the potential risks and benefits of antifungal prophylaxis [115, 116]. In populations with a low incidence, the disadvantages outlined above may outweigh the benefits of universal prophylaxis. Importantly, the incidence of IFD may vary based on climate, geography, and local epidemiology, highlighting the value of using both large multicenter trials and local institutional data to inform practices, just as we utilize local rates of antibacterial resistance to inform antimicrobial prescribing in the hospital [120]. Furthermore, when considering the application of antifungal prophylaxis to CAR T-cell therapy or to any novel immunotherapy, risk may differ by underlying disease (eg, hematologic malignancy vs autoimmune disease), timing of treatment (eg, second-line vs fifth-line therapy), and type of product (eg, allogeneic vs autologous). Continued assessment of these new applications of cellular therapy will be needed as well as a critical need to better characterize the risk factors associated with IFD in these novel populations, to better identify which patients may benefit most from targeted preventive strategies.

## PREVENTION OF INVASIVE FUNGAL DISEASE: APPLICATIONS TO CAR T-CELL THERAPY

**Figure 5.**
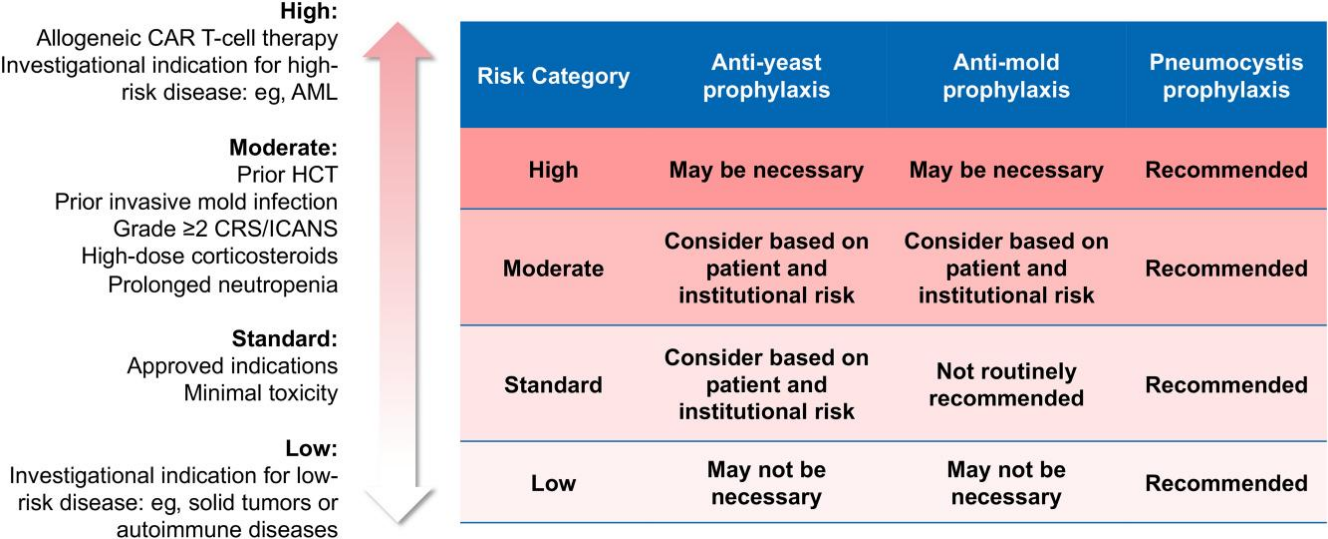
Framework for assessing the need for antifungal prophylaxis in chimeric antigen receptor (CAR) T-cell recipients with a focus on emerging novel indications for therapy. For currently approved disease indications, anti-mold prophylaxis may be utilized in some centers for patients with extended neutropenia (>20 d), high-dose or extended duration of corticosteroids (>3 d) for grade ≥2 cytokine release syndrome or immune effector cell–associated neurotoxicity syndrome, prior allogeneic hematopoietic cell transplantation, or a history of invasive mold infection. Some centers do not use any anti-mold prophylaxis, with acceptably low rates of invasive mold infection reported. The impact of other comorbidities such as chronic lung disease or diabetes mellitus is not currently known. Abbreviations: AML, acute myeloid leukemia; CAR, chimeric antigen receptor; CRS, cytokine release syndrome; HCT, hematopoietic cell transplantation; ICANS, immune effector cell–associated neurotoxicity syndrome.

There have been no prospective studies evaluating the use of antifungal prophylaxis or preemptive therapy following CAR T-cell therapy. Preventive strategies for IFD following CAR T-cell therapy vary widely and are primarily either adopted directly from clinical trial protocols or based upon expert opinion developed when the therapy was new and no data were available to guide rational use of prophylaxis. We suggest a risk-based framework to evaluate the need for antifungal prophylaxis as demonstrated in Figure 5.

Despite the variation in practice generally, there is consistency in the approach to PJP prevention, where prophylaxis is almost universally utilized, typically for at least 6–12 months [34]. The optimal duration remains unknown and several studies have demonstrated evidence of persistent T-cell depletion with low CD4 T-cell counts that extend beyond 1 year [57, 59]. CD4 cell counts may be a basic marker for patients with heightened long-term risk of opportunistic infections; however, late cases of PJP have been reported even in patients with normal CD4 counts, suggesting that the deficits in cell-mediated immunity are likely more complex [38]. In-depth exploration of long-term immunologic deficits may help to identify patients at higher risk and guide duration of PJP prophylaxis in a more precise manner.

Approaches to anti-yeast prophylaxis are less consistent across centers. Fluconazole or micafungin are given universally in some centers, while other centers employ a targeted strategy for those with prolonged neutropenia or receipt of corticosteroids (Table 3). Some centers do not administer any anti-yeast prophylaxis but utilize a protocol where patients receive micafungin in the setting of prolonged or recurrent neutropenic fevers [38]. All of these approaches appear to be reasonable since there is no indication that centers without universal anti-yeast prophylaxis have higher risk of invasive yeast infections including invasive candidiasis. The incidence of resistant yeast isolates thus far appears to be low, though breakthrough cases of *Candida krusei* and *Candida glabrata* have been reported in centers using fluconazole prophylaxis. Further study of targeted approaches to anti-yeast prophylaxis are needed, particularly as patients receive CAR T-cell therapy earlier in their disease state with fewer preceding lines of treatment.

The use of universal mold-active prophylaxis after CAR T-cell therapy is not currently supported by available data as the rate of IMI across all evaluated studies is low (Figure 5) [121]. In CD19 recipients, all studies since 2020 demonstrated particularly low IMI incidence (0–2%), which has precluded formal risk factor analyses. However, cases have been described in patients who have undergone prior allogeneic HCT, those who received prior Bruton tyrosine kinase inhibitor therapy, and in those who developed severe CRS/ICANS with high-dose corticosteroid use, all of which may independently increase the IMI risk, suggesting that these secondary risk factors may play a key role in identifying patients who could benefit from prophylaxis. The incidence of IMI among BCMA CAR T-cell recipients has been slightly higher in recent studies (up to 6% in 1 study of 32 patients), which could reflect the inclusion of clinical trial patients with heavy pretreatment. However, given the limited number of studies, further investigation of IMI risk after BCMA CAR T-cell therapy is needed. The current approach to mold-active prophylaxis is most often a targeted one, with mold-active azoles provided to patients with prolonged neutropenia or corticosteroids (Table 3), though some centers without any mold-active prophylaxis report acceptably low risk of IMI [38, 84]. At this point, it does not appear that universal mold-active prophylaxis is needed after CAR T-cell therapy and in fact could lead to avoidable toxicity, drug–drug interactions, and breakthrough infections that outweigh overall benefits. More targeted approaches based on individual risk stratification are reasonable and require a better understanding of IFD epidemiology in the CAR T-cell therapy setting. Optimal prevention strategies should be dynamically reevaluated as CAR T-cell therapy is administered as an earlier line of treatment in less immunocompromised populations or to novel oncologic and nononcologic populations. Prospective studies of prophylaxis strategies would contribute greatly to the field, and other areas in need of investigation include differentiation of risk factors for early and late IMI, which would also inform preventive strategies.

CAR T-cell therapy is rapidly expanding in 2 directions—to settings that may have a higher risk of IFD (eg, allogeneic CAR T-cell therapy, CAR T-cell therapy for AML) and to settings that may have a lower risk for IFD (eg, earlier line of treatment in onco-hematological indications, treatment of solid tumors or autoimmune diseases). Considering this, we must remain diligent in assessing the specific risks and epidemiology of IFD in the expanding CAR T-cell therapy population. Infectious diseases specialists need to play a key role in rigorous infection reporting and evidence-based decision-making around diagnosis, prevention, and management of IFD to improve patient outcomes and ensure antifungal stewardship.

## Notes

***Author contributions.*** J. S. L. conceptualized and co-wrote the manuscript, edited the manuscript, and created figures. E. K. co-wrote and edited the manuscript and created a figure. D. F., T. M., G. R. T., D. P. K., J. V., and J. W. B. reviewed and edited the manuscript. S. P. H. conceptualized, reviewed, and edited the manuscript.

***Data availability.*** The data that support the findings of this study are available from the corresponding author upon reasonable request.

***Patient consent.*** This is a review article and does not include original patient information or any factors necessitating patient consent.
